# Supplementary material for: From sequence to enzyme mechanism using multi-label machine learning
Source: BMC Bioinformatics. 2014 May 19;15:150. doi: 10.1186/1471-2105-15-150 (PMC4229970; doi:10.1186/1471-2105-15-150)
Supplement: Additional file 2 — Java code of ml2db. Additional file ml2db_code.tar.gz contains the Java source code to run the multi-label machine learning experiments and save the results to database. The code’s Javadoc is included. [file 1471-2105-15-150-S2.zip › additional file 2/ml2db/ml2db/doc/main/ml2db/mulan/mulanarff/MulanAttributeFactory.html]

MulanAttributeFactory


JavaScript is disabled on your browser.


- Overview
- Package
- Class
- Use
- Tree
- Deprecated
- Index
- Help

- Prev Class
- Next Class

- Frames
- No Frames

- All Classes

- Summary:
- Nested |
- Field |
- Constr |
- Method

- Detail:
- Field |
- Constr |
- Method


main.ml2db.mulan.mulanarff

## Class MulanAttributeFactory

- java.lang.Object
- - main.ml2db.weka.AttributeFactory
  - - main.ml2db.mulan.mulanarff.MulanAttributeFactory

- ---

    

  ```
  public class MulanAttributeFactory
  extends AttributeFactory
  ```

  In the Mulan ARFF, the class attribute is not generated. Instead, a list of
  class attributes were previously added to the attributes.

  Version:
  :   7 May 2010

  Author:
  :   Luna De Ferrari ldf(at)st-andrews.ac.uk

- - ### Field Summary

    - ### Fields inherited from class main.ml2db.weka.AttributeFactory

      `BINARY_ATTRIBUTE_ABSENT_VALUE, BINARY_ATTRIBUTE_PRESENT_VALUE, DUMMY_VALUE, NOMINAL, NUMERIC, UNKNOWN`
  - ### Constructor Summary

    Constructors

    Constructor and Description

    `MulanAttributeFactory(MulanDataSetGenerator dataSetGenerator)`
  - ### Method Summary

    Methods

    | Modifier and Type | Method and Description |
    | --- | --- |
    | `java.util.TreeMap<java.lang.String,java.lang.String>` | `getAttributesNameTypeMap()` |
    | `java.util.TreeSet<java.lang.String>` | `getClassAttributes()` Get the Mulan class attributes |
    | `MulanDataSetGenerator` | `getDataSetGenerator()` |

    - ### Methods inherited from class main.ml2db.weka.AttributeFactory

      `attributeIsValid, createAttributes, generateBinaryAttribute, generateNominalAttribute, generateNominalAttributeWithDummy, generateNumericAttribute, getAttributeAbsentOption, getAttributeByName, getAttributePresentOption, getAttributes, getAttributesNamesList, getBinaryAttributeValuesSet, getClassAttribute, getNameAttributeMap, getZeros, intialise, setAttributesNameTypeMap`
    - ### Methods inherited from class java.lang.Object

      `equals, getClass, hashCode, notify, notifyAll, toString, wait, wait, wait`

- - ### Constructor Detail


    - #### MulanAttributeFactory

      ```
      public MulanAttributeFactory(MulanDataSetGenerator dataSetGenerator)
      ```
  - ### Method Detail


    - #### getAttributesNameTypeMap

      ```
      public java.util.TreeMap<java.lang.String,java.lang.String> getAttributesNameTypeMap()
      ```

      **Overrides:**
      :   `getAttributesNameTypeMap` in class `AttributeFactory`


    - #### getClassAttributes

      ```
      public java.util.TreeSet<java.lang.String> getClassAttributes()
      ```

      Get the Mulan class attributes


    - #### getDataSetGenerator

      ```
      public MulanDataSetGenerator getDataSetGenerator()
      ```

      **Overrides:**
      :   `getDataSetGenerator` in class `AttributeFactory`


- Overview
- Package
- Class
- Use
- Tree
- Deprecated
- Index
- Help

- Prev Class
- Next Class

- Frames
- No Frames

- All Classes

- Summary:
- Nested |
- Field |
- Constr |
- Method

- Detail:
- Field |
- Constr |
- Method
